# Editorial: Understanding and promoting factors which affect healthy ageing: physical activity, sleep patterns and nutritional habits

**DOI:** 10.3389/fpubh.2024.1464360

**Published:** 2024-09-12

**Authors:** Radenko M. Matic, Stevo Popovic, Juel Jarani, David Paar

**Affiliations:** ^1^Faculty of Sport and Physical Education, University of Novi Sad, Novi Sad, Serbia; ^2^Western Balkan Sports Innovation Lab, Podgorica, Montenegro; ^3^Faculty for Sport and Physical Education, University of Montenegro, Niksic, Montenegro; ^4^Faculty of Movement Sciences, Sports University of Tirana, Tirana, Albania; ^5^Faculty of Health Sciences, University of Pécs, Pécs, Hungary

**Keywords:** physical fitness, sleep quality, nutrition, wellbeing, mental health

## Introduction

Modern problems caused by an inactive lifestyle represent the general framework of civilization. The obesity epidemic, based on the latest NCD-RisC study of 2024, revealed that more than 1 billion people in the world are now living with obesity, about 880 million adults and 159 million children (1). From a global perspective, factors associated with multiple risk behaviors are linked to the future development of non-communicable diseases (NCDs) (2). The issue of healthy aging is a topic that needs interdisciplinary perspectives. Further progress in adopting an active lifestyle requires an analysis of the modern man's lifestyle and changes in his/her daily routines and habits at work, during free time, in performing some physical activity, in sleep and in nutrition. These changes occur under the influence of technological progress but also unexpectedly (as in the case of the COVID-19 pandemic). Synchronizing circadian clocks requires a broader discussion about physical activity, sleep, and nutrition to identify novel disease interventions (3). Raising awareness of healthy lifestyle habits could significantly reduce costs, so governments should consider and incorporate them into various regulations and policies (4). In addition, sleep patterns are considered a vital factor in promoting health, with research highlighting the importance of addressing sleep disturbances to reduce the risk of medical illnesses such as cardiovascular disease, cancer, and depression (5).

Therefore, it is necessary to explain, learn about, and understand the mechanisms of action of factors that influence healthy aging to monitor their changes and to promote adequate knowledge at different levels of action, from preventive to corrective actions, in the broader social community. Such knowledge can provide a better understanding of the importance of all individual factors and their integral context and facilitate the promotion and active action of various social interventions.

This Research Topic provides significant findings that could have a profound effect on the promotion of social interventions to activate the principles of active lifestyles and healthy aging throughout one's lifespan.

## Contribution to the field

The present Research Topic (RT) has been recommended as a holistic approach to addressing the challenges of healthy aging (HA). The articles in this RT covered physical activity, sleep patterns, and dietary habits as critical contextual factors that affect healthy aging. This editorial presents a unique form of scientific reasoning regarding HA by examining the social environment and community issues in context. The purpose of this RT is to propose contemporary knowledge for understanding and promoting HA. As the primary outcome, this RT consists of 24 articles. All of the published papers tried to enlighten the readers about this multidisciplinary topic from different perspectives. The Research Topic with the theme “*Understanding and promoting factors which affect healthy ageing: physical activity, sleep patterns, and nutritional habits”* is an attempt by the editors to use a holistic approach to integrate all essential human routines during everyday life in the context of healthy aging. This Research Topic provided an opportunity to gather insights from different perspectives, and the articles compiled offer a scientific argumentation that summarizes four key topics: (1) active lifestyle and healthy aging; (2) physical activity and sleep patterns—napping, sleep, and cognitive health; (3) physical activity, sleep patterns, and cognitive function, and (4) social environment and community factors—community-based care, dietary habits, and public health strategies.

### Active lifestyle and healthy aging

First, Wu D. et al. presented valuable findings emphasizing the importance of maintaining a healthy lifestyle to slow biological aging. In addition, He, Le et al. identified methods for selecting markers of aging (physiological, biochemical, and molecular indicators) and established the framework for screening such markers. An examination of the meta-analysis by Sun et al. about the effects of exercise training on increasing skeletal muscle iNAMPT levels showed that exercise supports cellular energetics and overall health span. The authors suggest that exercise should be recommended as a natural slow-aging approach. Continuing in the context of the findings from their previous research, Wang X. et al. identified three different lifestyle categories in older adults: relatively positive, mixed, and relatively negative. Each type of improvement in these categories increases the physical, mental, and self-rated health of older adult respondents. The authors suggested that older adults adopt a positive lifestyle with all the advantages of their intelligent model. As a good tool for maintaining a healthier lifestyle, Martín et al. emphasized the use of fitness apps in multiple facets of people's everyday routine activities.

Lima de Oliveira et al. confirmed that the level of physical activity and sedentary behavior are independent predictors of mortality. Hypertension, a leading risk factor for mortality, requires a multicomponent management approach. Thus, Zhang et al., conducted China's Health and Retirement Longitudinal Study, and used a Bayesian network model to determine the factors related to heart attacks complicated with hypertension (HAH). Results from a large data sample showed that age, sleep duration, physical activity, and self-reported variables were directly associated with HAH. Based on these findings, the authors concluded that the Bayesian network model offers valuable information about HAH risk with possible implementation in monitoring this health problem. Based on the findings, from both studies, the level of physical activity can be covered by the regular daily routine and by performing some housework to preserve the physical function in older people. Furthermore, Xiao et al. recommended ancient methods as tools to fight cardiovascular diseases. The authors advocate for a “zero–one–all things” approach, where “zero” represents physical inactivity and hypertension, and “one” refers to an “inclusive and culturally appropriate exercise training cocktail.” In this context, “all things” represents the approach of an active lifestyle and healthy aging.

### Physical activity, mental health, and sleep patterns—Napping, sleep, and cognitive health

Several authors have tried to explain the relationship between physical activity, physical frailty, and depressive symptoms in adults aged 60 years and older. Wang Y. et al. concluded that gender differences exist in these types of relationships. From a male perspective, findings on grip strength and gait ability could be quality indicators of frailty for predicting depressive symptoms. From a female perspective, the results showed that physical activity is very beneficial. As a good strategy to prevent depression in older adults (≥65), Chen et al. recommended collecting a higher amount of moderate-to-vigorous than light physical activity during the day. Similarly, accumulating more daily MVPS can reduce the fear of falling in community-dwelling older women, which Wu S. et al. suggested in their research. In this process, physical fitness plays an essential role.

This topic was further pursued with a systematic review by Sadaqa et al., who presented valuable evidence on the effectiveness of exercise interventions for fall prevention in community-dwelling older adults. The authors strongly recommend that these interventions include physical exercise to improve balance, lower extremity strength, and mobility. Furthermore, Wu J. et al. also investigated the positive effects of square dance interventions on sleep quality in middle-aged and older Chinese women. These effects are based on the mediation of social support and depressive symptoms. Dózsa-Juhász et al. reported a relationship between premenstrual syndrome and regular physical activity, mental state, and stress. It is important to mention that the authors confirmed the negative correlation between regular physical activity and perceived stress levels.

Finally, Yu et al. proposed specific leisure time activities beneficial to the mental health of adults. One such activity recommended by the authors is Guangchangwu, a special characteristic of Chinese culture.

### Physical activity, sleep patterns, and cognitive function

Several authors have contributed to the understanding of the interrelations among physical activity, sleep patterns, and cognitive function. For instance, Tao et al. concluded that adequate sleep and physical activity can affect cognitive performance. This relationship is part of a holistic, healthy lifestyle that we strive to understand. Kimura et al. also made significant contributions, highlighting the negative relationship between poor sleep quality and lower levels of physical activity and cognitive ability in older adults. Their findings have led to the development of new intervention approaches. In the same vein, Wu H. et al., conducted a longitudinal study and confirmed that non-nappers have progressive odds of cognitive decline. This collaborative research is shaping our understanding of healthy aging and reducing the odds of cognitive decline.

Finally, Milot et al. developed a study protocol to evaluate the efficacy of multimodal home-based videoconferencing interventions on sleep in older adults. The authors proposed four subsamples (25 participants per group) of a randomized controlled trial: (1) a physical exercise training group, (2) a physical exercise training combined with a bright light exposure group (*n* = 25), 3 a galvanic vestibular stimulation group (*n* = 25) or a control (i.e., health education) group (*n* = 25). The expected results may support the development of strategies to improve sleep quality in older adults.

### Social environment and community factors—Community-based care, dietary habits—Lifelong nutrition and public health strategies

The social environment and community factors can be significant strengths or barriers to healthy aging. In this context, we should consider many sociodemographic characteristics (gender, age, education, social, and marital status) that explain the time spent on physical activity, as supported by the findings of Trujillo-Barberá et al.. Wang Y. et al. considered that the high quality of home care services for older adults (≥60 years) is determined by factors such as age, marital status, pre-retirement occupation, source of financial resources, and mode of residence. Further progress has been made in addressing emotional needs, reducing the costs of aging, and addressing balanced and well-integrated healthcare issues. Similarly, He, Wei et al. confirmed the quality of community home-based aged care care services. The authors opened the discussion on government health expenditures and recommended the establishment of a socialized aged care system in China. Finally, the research by Casals et al. provided practical evidence on the effects of educational and related programs on improving physical function, sleep patterns, and nutritional status of older adults. The authors concluded that this educational program could significantly promote an active lifestyle in vulnerable populations.

## Conclusion

The diversity of approaches taken by the authors in researching the topics of this Research Topic confirms the complexity of establishing essential life principles and adopting active lifestyle strategies in the context of healthy aging. The use of the collected knowledge and its application at the family, school, and social levels offers the potential to improve each of the examined factors (physical activity, sleep characteristics, and eating habits) both separately and integrally. Such applications can contribute to the spread of a positive influence on the determinants of everyday life and its environmental and community factors. Finally, following the ongoing efforts to improve legal regulations and conditions at the community level, a significant focus is on the impact of promoting healthy aging.
